# Sensitivity and reliability of screening measures for paternal postpartum depression: an integrative review

**DOI:** 10.1038/s41372-021-01265-6

**Published:** 2022-01-01

**Authors:** Erin Kennedy, Kristen Munyan

**Affiliations:** School of Nursing, Oakland University, Oakland, MI USA

**Keywords:** Population screening, Depression

## Abstract

The American Academy of Pediatrics (AAP) recommends screening mothers for Postpartum Depression (PPD) during the postpartum period. Research shows depression in parents is associated with impaired growth and development in their children. The National Perinatal Association (NPA) encourages screening fathers for depression at least twice during the first postpartum year, however a preferred screening tool has yet to be determined. To promote optimal outcomes for children, providers must assess the mental health of all new parents, regardless of gender. Therefore, the purpose of this integrative review is to examine previous scientific evidence regarding the sensitivity of screening measures for postpartum depression in fathers. Future research should be directed towards describing the psychometric properties of a tool to assess postpartum mood disorders in American fathers while analyzing appropriate screening intervals during the postpartum period.

## Introduction

The scientific literature consensus is that paternal postpartum depression (PPD) is a real phenomenon affecting new fathers. Healthcare providers should incorporate screening for paternal depressive symptoms during healthcare encounters, particularly in the pediatric setting [1]. Despite this agreement and a recent call to action issued by the American Academy of Pediatrics [2], clinicians wishing to integrate this recommendation into their practice may find it challenging to locate clear guidelines for implementing screenings.

Empirical work completed over the past decade related to the concept of PPD suggests that screening is an essential aspect of optimal care for the entire family dynamic. However, it is unclear which screening tool should be utilized to assess depression in men during the postpartum period. A greater understanding of screening tools focused on assessing depression in fathers is needed. Therefore, this integrative review aims to examine previous scientific evidence regarding the sensitivity of screening measures for PPD in fathers to inform future research regarding PPD prevalence among new fathers in the United States.

## Background

Paternal PPD affects fathers globally; however, it’s rarely discussed, and no current research focuses on American fathers’ screening. Previous research shows that as many as 1 in 10 fathers develop paternal PPD, with the risk rising 25–50% for fathers whose partners suffer from PPD [3]. Prevalence studies have varied considerably based on the populations surveyed and the screening instrument or measurement method used, with an overall estimated prevalence of 8.4% [4]. According to the Centers for Disease Control and Prevention, about one in eight mothers will experience PPD symptoms nationally. With these estimates varying by state, rates are seen as high as 1 in 5 [5]. Paternal PPD has developed prenatally or within the first 12 months of the infant’s life, with the highest prevalence of PPD between 3 and 6 months after birth [3]. This timeline is later than what is traditionally seen in mothers who develop PPD.

Clinical manifestations of paternal PPD are similar to maternal symptoms, however men experience additional symptoms that may require individualized screening criteria that are not currently included in the Edinburgh Postnatal Depression Scale (EPDS). The EPDS is a 10-item scale addressing predominately depressive symptoms (8 items) and some symptoms of anxiety (2 items) [6]. The EPDS has been extensively studied and has been found to have adequate psychometric properties for the detection of PPD in women with a 94% sensitivity at a cut-off at 11 [7]. Validation studies have utilized various threshold scores in determining which women were positive and in need of referral [2].

Screening tools developed for the identification of depression in women may not adequately capture depressive symptoms in males. In assessing fathers, additional symptoms to consider include aggressiveness, substance misuse, risk-taking behavior, partner violence, and infidelity [8]. Paternal PPD can affect both parents’ ability to bond with their infant. A father’s depressive symptoms can directly inhibit the mother’s ability to bond with the infant, potentially causing additional complications with newborn care such as breastfeeding and attachment [8]. Literature has shown that fathers with depressive symptoms in the first 6 weeks of the postpartum period went on to experience impaired bonding with their infant at 6 months postpartum [9].

Risk factors for PPD are similar for mothers and fathers, however additional known risk factors exist for fathers that should warrant purposeful screening [8]. Risk factors for PPD, more commonly seen in fathers, is witnessing delivery complications, specifically life-threatening or those that result in a poor outcome [8]. A history of severe depression or increased symptom scores for depression and anxiety prenatally were the strongest predictors of paternal depression in the postpartum period [9]. One of the most significant risk factors for paternal PPD is the depression status of the father’s partner [1]. Partners had experienced an increase in depressive symptoms when their partners were also being affected by PPD symptoms. However, those who had a partner not affected by PPD had quicker resolution of their PPD symptoms.

Research shows depression in parents is associated with impaired emotional development in their children [10, 11]. Further studies have indicated paternal depression is related to several poor developmental outcomes for children, including acting out behaviors, diagnosis of pediatric psychiatric disorders, defiant/conduct disorders, and disruptions to partner relationships [10, 11]. Children are two to four times more likely to develop depression before adulthood when they have parents who have suffered from depression [10, 11].

The National Perinatal Association (NPA) encourages screening fathers for depression at least twice during the first postpartum year, however there is not an established recommendation for a screening tool in this population. The United States Preventative Task Force offers no formal recommendations on paternal depression screening to guide clinical practice [12]. Care should be directed towards preventing and treating paternal PPD before the neonate’s birth and continued attention to screening throughout the postpartum period [13]. Thus, the purpose of this integrative review is to describe the published literature regarding the psychometric properties of existing screening tools that could be used for detecting depression in men during the postpartum period.

## Methods

The authors used Whitmore and Knafl’s (2005) five-stage approach for integrative review construction to guide the literature search [14]. Stages include problem identification, literature search, data evaluation, data analysis, and results presentation.

### Problem identification

The purpose of this integrative review is to examine previous scientific evidence regarding the psychometric properties of screening measures for PPD in fathers.

### Literature search

CINAHL Complete, EBSCO Academic, and PsycINFO were searched for articles relevant to the analysis of existing screening measures for paternal PPD. Articles were limited to those published in English and published in a peer-reviewed publication prior to September 2021. The search resulted in 256 titles in the initial yield. Keywords: Father* OR paternal OR dad* OR male AND postpartum depress* OR postnatal depress* OR depress* AND screening tool OR assessment tool OR instrument. Variations in spelling such as postpartum and post-partum were also used. After the authors removed 62 duplicates, they reviewed 194 article abstracts. The search located an additional six articles through archive searching. The authors reviewed abstracts together, applied inclusion and exclusion criteria, resulting in *n* = 18 included articles for review in full text. Of the 18 articles reviewed in full-text, the authors eliminated three as the aims were to evaluate an intervention rather than the screening instrument, three were found to be reporting on prevalence rather than on psychometric properties of the instruments used, one involved self-screening rather than provider-based screening and one compared positivity rates across instruments but did not complete analysis of the psychometric properties of the instruments. The total included articles in the integrative review were *n* = 10.

#### Inclusion criteria

Articles reporting on the psychometric properties of the screening measure used to detect PPD in men, published in English, and peer-reviewed.

#### Exclusion criteria

Articles reporting only prevalence data, unpublished dissertation work, conference abstracts/proceedings, articles not published in English, and articles not peer-reviewed.

**Fig. 1:**
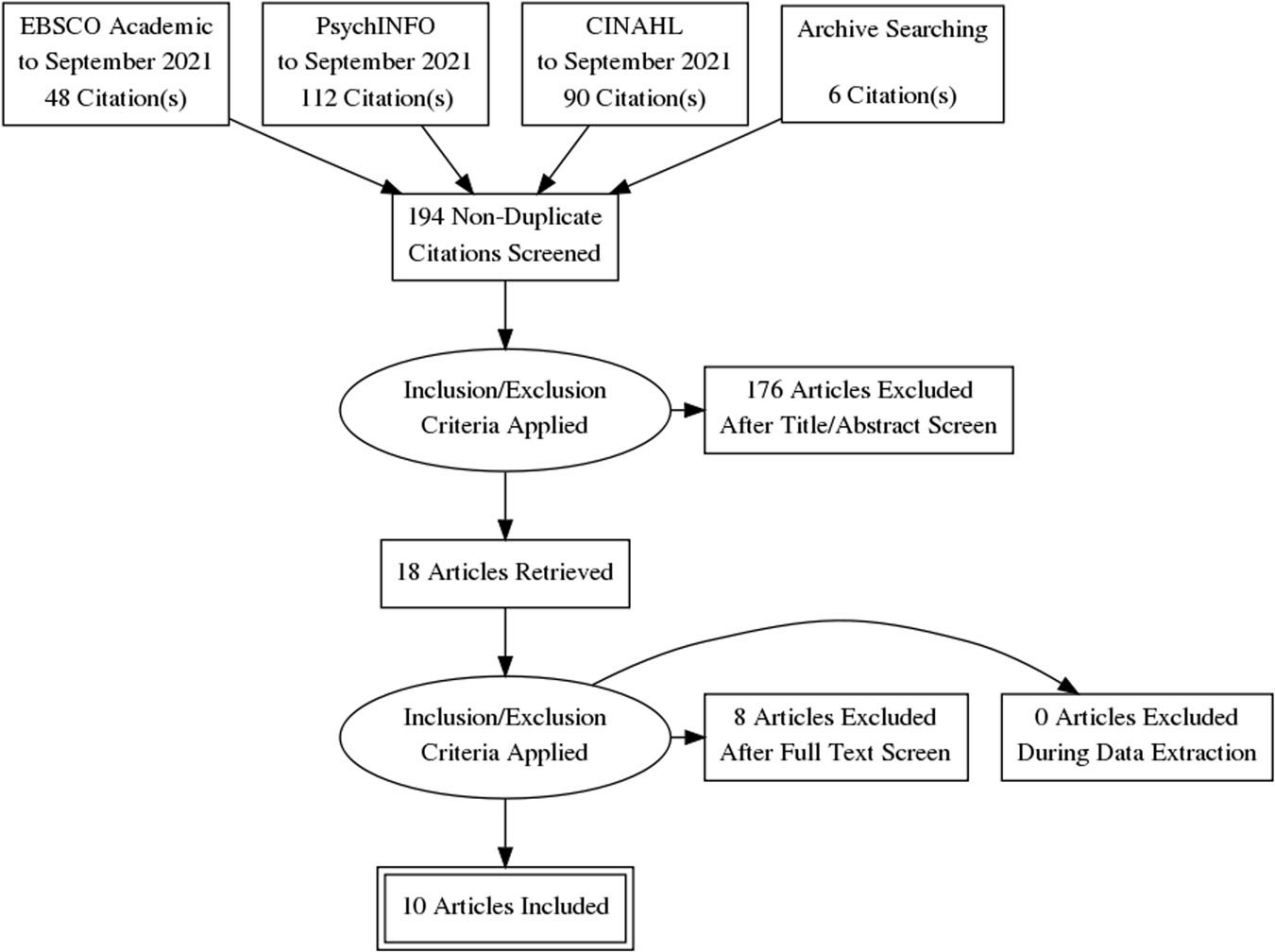
Summary of evidence search process and selection. Selection process for studies included is in compliance with Preferred Reporting Items for Systematic Reviews and Meta-Analyses (PRISMA).

### Data evaluation

Included studies are summarized and presented in Table 1 for ease of reference. Of the included studies, three were conducted with the participation of Swedish fathers, one with Chinese fathers, one with Vietnamese fathers, one with Japanese fathers, one with UK fathers, one with Saudi fathers, one with Australian fathers, and one with Italian fathers. All of the involved studies evaluated the EPDS. Several included analyses with correlation to demographic factors [15–17]. Duration from birth to screening was variable across studies and ranged from 4 weeks to 6 months. No study examined the relationship between time of screening and likelihood of positivity.

### Results presentation

Table 1

**Table 1:** Summary of literature.

Author (date)	Study stated purpose	Subjects/setting	Scale analyzed	Methodology	Results	Clinical implications
Carlberg et al. (2018)	Compare depression assessment and demographic factors of the EPDS and GMDS.	*n* = 3656 Swedish fathers	EPDS and GMDS items, the survey also contained questions covering possible risk factors related to the sociodemographic variables.	Cross-sectional design, surveys sent via letter with recruitment list being obtained through tax records.	Results suggest that neither scale alone is sufficient for depression screening in new fathers, and the decision of EPDS cutoff is extremely important.	Neither the EPDS or the GMDS may be adequate for screening. Clinician’s should be conservative in determining EPDS cut-offs when screening men for postnatal depression.
Edmondson et al. (2010)	Establish a reliable cut point for the EPDS for UK fathers and to determine its reliability by comparing it to structured clinical interviews.	Couples recruited 7 weeks after birth. Questionnaire response of couples agreed to a home visit and SCI.	EPDS, SCID, demographic characteristics.	EPDS at 7 weeks after the birth of their child. SCI conducted to correlate positive screening to SCID findings.	Fathers with depression scored higher on the EPDS than non-depressed fathers.	The EPDS had acceptable sensitivity and specificity at a cut off score of over 10. EPDS may be useful for perinatal screening for depression in men.
Lai et al. (2010)	Compare the psychometric properties of the EPDS, BDI, and PHQ-9.	*n* = 551 men, 8 weeks postpartum, in Hong Kong.	EPDS, BDI, and PHQ-9, validated against the SCID.	Collection of demographic data occurred immediately postnatal prior to discharge. Participants sent survey screenings at 8 weeks postpartum. Men with positive screenings invited to participate in SCID.	The EPDS was significantly more accurate at detecting postnatal depression in Chinese men than the BDI or PHQ-9.	The EPDS was recommended for postnatal depression with a cutoff score of over 10/11.
Loscalzo et al. (2015)	Contribute to the validation of the EPDS on a sample of Italian fathers and conduct factorial analysis.	Two samples. First, *n* = 334 fathers, 39 depressed new fathers. Second, *n* = 102 fathers, 22 depressed new fathers.	EPDS, the BID, and the CES-D. Demographic questions such as age, education level, and marital status.	Conducted explorative factor analysis and receiver operator characteristic analysis using samples with new fathers known to have depression.	EPDS did not appear sensitive to depression but rather to symptoms of depression and distress.	The EPDS is sensitive for screening for perinatal distress and identifying fathers in need of emotional support.
Matthey et al. (2001)	To validate the EPDS for use in fathers and to establish an acceptable cut off point.	*n* = 230 mothers and *n* = 208 fathers recruited to be diagnostically interviewed and administered the EPDS at 6 weeks postpartum.	EPDS, diagnostic interviewing.	EPDS scores were compared to caseness established with diagnostic interviewing. Item analysis was also conducted.	EPDS was valid and reliable for screening mood disorders in fathers, but item analysis revealed that fathers were significantly less likely to answer affirmatively to seven items than mothers.	Recommended a cutoff point of 5 to 6 for detection of mood disorder (anxiety or depression) in men.
Massoudi et al. (2013)	Investigate the accuracy of the EPDS for detecting anxiety and depression in new fathers. Compare the factor structure in fathers versus mothers. Validate the Swedish version of the EPDS in relation to DSM-IV criteria for major and minor depression.	*n* = 1014 couples were sent the EPDS and the HAD-A subscale 3 months postnatally. Fathers who scored high were asked to participate in a diagnostic interview to assess for depression or anxiety disorder.	EPDS and the anxiety subscale of the HAD scale. Questions dealing with the participants’ age, current occupation, education, native language and number of children were also included in the questionnaire.	Screening survey and structured clinical interviews were used for data collection.	The EPDS yields high sensitivity and specificity, but low positive predictive value when screening for probable major depression at the optimal cut-off score of 12 or more.	The EPDS was more sensitive to distress than depression in postnatal fathers. EPDS may be useful in screening for major depression, but those with minor depression may be missed. Neither the EPDS or HAD-A scale was recommended for use in screening for anxiety.
Nishimura et al. (2010)	This study investigated risk factors of depression in Japanese fathers at 4 weeks post‐partum using a cross‐sectional design.	Responses were obtained from mothers and fathers. There were *n* = 146 fathers who completed both the EPDS and the CES‐D Scale, *n* = 133 analyzed.	The EPDS and the CES-D were used to assess depressive symptoms.	Mothers at the 1-month postnatal check were recruited at two general hospitals and two private clinics. The two surveys were sent to the mothers and fathers.	No association between paternal and maternal depression at 4 weeks post‐partum. Paternal depression was associated with employment status, a history of receiving psychiatric treatment, and unintended pregnancy.	Providers should independently screen for depression in fathers and mothers. Additional research is needed to clarify the specific risk factors for postnatal depression in fathers.
Psouni et al. (2017)	The study had multiple aims: Investigate depressive symptoms in new fathers postnatally. Test a modified EPDS scale using items from the GMDS for an increase or decrease in sensitivity.	*n* = 438 cases were reviewed.	Demographic and circumstantial variables such as stressful life events, age of the father and child, occupation, education level, income, number of children, and whether the father and/or partner had previously, and at the time of the study, received professional help for mental health problems, were all examined.	Fathers were surveyed online using the BDI and GMDS scale initially. Fathers were also surveyed using a modified EPDS that incorporated items from the GMDS.	The modified EPDS with GMDS items had greater sensitivity than the EPDS alone.	Existing scales may be insufficiently sensitive to detect postnatal depression among new fathers. Additional research is needed.
Shaheen et al. (2019)	To determine the cutoff for use of EPDS for Saudi fathers and to estimate PPD prevalence.	*n* = 290 Saudi fathers who took the EPDS, *n* = 72 of which were invited to participate in SCID. Fathers were recruited by random sample from those visiting the birth registration office.	EPDS and demographic data, SCID.	Cross-sectional study with a subsample participating in further diagnostic interviewing.	The authors found a cutoff score of 8/9 was optimal to achieve sensitivity of 77.8% and specificity of 81.3%.	Adjusted prevalence was 16.6%. Authors emphasized need to screen men during the postpartum period.
Tran, et al. (2011)	To validate three existing scales for use in screening men in Vietnam for common mental health concerns in the perinatal period.	*n* = 231 partnered Vietnamese men were recruited.	EPDS, Zung SAS, GHQ12, SCID, modules for depression, generalized anxiety, and panic disorder, Vietnamese translations and cultural verification were given.	Translations of EPDS, Zung SAS, and the GHQ-12 were validated against SCID. Post-hoc analyses, Receiver Operating Characteristic (ROC) analyses, and Cronbach’s alpha were conducted on each scale.	While all measures had acceptable reliability, the sensitivity of the EPDS in men was significantly lower than in women.	The authors recommended that appropriately translated copies of each instrument be available to local primary care offices to conduct screenings with new fathers.

## Findings

Included articles were reviewed in full text by both authors and compared notes to identify common discussion points across studies. Themes of cultural variations, demographic influences, and the concept of distress versus depression were identified through this process and are explored below.

Of the included studies, there was considerable variation in measurement methods and instruments used. All of the studies (*n* = 10) included the EPDS. This review found significant variability in cut-off scores used and sensitivity and specificity among populations (See Table 2). Other instruments included the Gotland Male Depression Scale (GMDS), which consists of 13 items and addresses typical male depressive symptoms, including aggression and irritability [18]. Two studies used either complete or portions of the GMDS. Neither found that it was independently sufficient to screen males in the postpartum period [15, 19]. While the Beck Depression Inventory (BDI), a scale used for adults across the lifespan, was employed in three of ten studies, only the article by Lai et al. reported sensitivity (100%) and specificity (81%) of the instrument [17]. In the studies, Tran et al., Edmondson et al., and Lai et al., structured clinical interviewing (SCID) for DSM diagnosis was used to compare caseness with the results of other screening measures [16, 17, 20]. Matthey et al. validated EPDS scores in fathers against their maternal partners with a cut point score of 5/6, a relatively low cut point compared to subsequent studies [21]. Among the studies reviewed there was not an optimal cut point determined.

### Cultural variations

Masoudi et al. tested a subscale of the EPDS and the HAD-A, the Anxiety subscale of the Hospital Anxiety and Depression Scale [22]. The HAD Scale is a 14-item scale assessing both anxiety and depression [22]. It was determined that it would have little practical significance in identifying fathers with probable anxiety disorders during the postnatal period. Recommendations included a cut-off score of 12 or more to yield high estimated sensitivity and specificity. These findings are similar to Carlberg et al., who also assessed Swedish fathers [15]. However, Carlberg et al. determined that the fathers scoring 10 or 11 on the EPDS (5.2%) constituted a substantial group of potentially depressed fathers who would not be detected if a 12 or higher score is utilized for the EPDS [15]. Psouni et al. combined the EPDS and the GMDS questions and completed an item reduction to leave a 12-question instrument. They then tested the shortened instrument on Swedish fathers and compared it to the original instruments [19]. Psouni et al. sensitivity and specificity may not have reflected the same results as other Swedish studies. However, given men’s low help‐seeking behavior, improved sensitivity in screening instruments is still imperative for prevention and treatment for fathers with depressive symptoms [19]. Psouni et al. addressed both traditional and male-specific depressive symptoms, with attentiveness to commonly known symptoms among fathers [19]. In Shaheen et al. Saudi fathers were found to have a prevalence of depression off 16.6% and the EPDS cut point was recommended at 8–9 [23]. This variability in the reported analysis is consistent with the demographic data. For a complete analysis of cut points, sensitivity and specificity, see Table 2.

**Table 2:** Summary of psychometrics with cultural variations in findings/recommendations.

Article/Author	Scale	Population	Cutoff points	Sensitivity (Se) and specificity (Sp)
Carlberg et al. (2018)	EPDS GMDS	Swedish Fathers	EPDS 10/12 or more and/or 13 or more on the GMDS	Se or Sp not reported. Cronbach’s α measured internal reliability for EPDS (0.83) and GMDS (0.88). The intercorrelation between the EPDS and GMDS was assessed by Pearson’s test (0.76, *p* < 0.001).
Edmondson et al. (2010)	EPDS	UK Fathers	EPDS ≥ 10	EPDS: Se 89.5% and sp 78.2% Analysis rerun using expanded database with more participants scoring low on the EPDS, the ≥10 cut off yielded a Se of 77.3% and Sp of 92.9%.
Lai et al. (2010)	EPDS PHQ-9	Chinese Fathers	EPDS 10/11 PHQ-9 3/4 BDI 5/6	EPDS: Se 91% and Sp 97%, positive predictive value 57%, and negative predictive value 99%. PHQ-9: Se 85% and Sp 81%, positive predictive value 23%, and negative predictive value 98%. BDI: Se 100%, Sp 81%, positive predictive value 21%, and negative predictive value 100%.
Loscalzo et al. (2015)	EPDS BDI CES-D	Italian Fathers	EPDS 12/13	EPDS: Se 90% and Sp 90%
Massoudi et al. (2013)	EPDS HAD-A	Swedish Fathers	EPDS major depression 12 or more, major or minor depression 9 or more HAD 4 HAD 8	EPDS 12: Se 100% (CI 63–100%), Sp 94.9% (CI 90–99%) (both values weighted) and a positive predictive value of 20.0% EPDS 9: Se 66.0% (CI 52–74%), Sp 86.3% (CI 78–94%) and a positive predictive value of 23.8% HAD 4: Se 51% HAD 8: Se 23.3%
Matthey et al. (2001)	EPDS CES-D Diagnostic interviewing	Australian Fathers	EPDS 9/10 (anxiety 5/6) CES-D	EPDS 9.5: Se 71.4%, Sp 93.8%, positive predictive value of 29.4%
Nishimura et al. (2010)	EPDS CES-D	Japanese Fathers	EPDS 7/8 CES-D ≥ 16	EPDS 7/8: Se 81.8%, Sp 94.1%, indicating ≥8. The optimal EPDS cut‐off score was determined at the maximum sum of Se and Sp.
Psouni et al. (2017)	EPDS GMDS EGDS BDI-II	Swedish Fathers	EGDS ≥ 14 BDI-II ≥ 14 cut-off for mild depression, 20 cut-off for moderate depression	EGDS: Se 90.5% and Sp 80.5% at cutoff point of 9
Shaheen et al. (2019)	EPDS SCID	Saudi fathers	EPDS 8/9 (anxiety 4 or more)	EPDS: Se 77.8%, and Sp 81.3%
Tran et al. (2011)	EPDS Zung SAS GHQ12	Vietnamese Fathers	EPDS 4/5 Zung SAS 35/36 GHQ-12 0/1	EPDS:Se 68.3% and Sp 77.4% Zung SAS: Se 70.7% and Sp 79.0% GHQ-12: Se 75.6% and Sp 74.7%

*BDI* Beck Depression Inventory-II, *CES-D* Center for Epidemiological Studies Depression Scale, *EGDS* Edinburgh Gotland Depression Scale, *EPDS* Edinburgh Postnatal Depression Scale, *GHQ12* General Health Questionnaire 12 items, *GMDS* Gotland Male Depression Scale, *HAD* Hospital Anxiety and Depression Scale, *HAD-A* Anxiety subscale of the Hospital Anxiety and Depression Scale, *PHQ-9* Patient Health Questionnaire, *SCID* Structured Clinical Interview for DSM-IV Axis II Personality Disorders, *Zung SAS* Zung’s Self-rated Anxiety Scale.

### Demographic influences

Of the ten studies, few examined the correlation between demographic characteristics and positive scores on depression screening instruments. Carlberg et al. found that fathers who had completed education levels of high school years three or four were significantly more likely to have depressive symptoms than those who had higher levels of education [15]. Carlberg et al. note that an established body of evidence acknowledges the correlation between poor school performance and academic achievement, and postulates that this may be why fathers with fewer years of education may be more likely to experience PPD [15]. Tran et al. reported that the cutoff point recommended in their analysis of screening scores from their population of Vietnamese manual labor workers was significantly lower than had been previously used in more affluent populations, implying a correlation between socioeconomic status and risk for PPD [16]. Lai et al. screened Chinese men, of whom only 56% had completed secondary schooling [17]. This study was consistent with Tran et al. in using a cut point of 9/10 to achieve optimal sensitivity.

Carlberg et al. performed the most extensive analysis of demographic factors, including comparing the factors associated with increased risk of positive scoring on the EPDS and the GMDS [15]. The authors noted that fathers screened using the EPDS were less likely to score positively if they had just one child. Fathers who had three or more children were more likely to score positive using the EPDS. Of fathers who were screened using the GMDS, being single, living separately from their partner, or being a widower was correlated with positive scoring. Carlberg’s work suggests that clinicians screening at-risk populations may benefit from further identification of demographic factors that contribute to the sensitivity of the instruments being used to screen for PPD in fathers.

Nishimura et al. investigated risk factors for depression in Japanese fathers at 4 weeks postpartum using the EPDS and the Center for Epidemiological Studies Depression Scale (CES-D) [24]. The CES-D is a 20-item scale that asks the individual to rate how often they experienced symptoms associated with depression over the past week [24]. The study found no correlation of a depressive condition between the mothers and fathers; however, they could identify risk factors for paternal depression. The identified risk factors included a history of psychiatric treatment (*p* < 0.01), unintended pregnancy (*p* < 0.01), temporary employment or unemployment (*p* < 0.001), additionally, retirement from work, major business readjustment, being fired from work, and changing to another job were significantly more likely in depressed fathers (*p* < 0.05) [24]. Nishimura et al. determined further identification of risk factors that directly influence employment status should be considered.

Shaheen et al. sample of Saudi fathers reported largely positive family and marital support and there were no significant differences noted between fathers with or without depression in regard to reported family, marital and work-related demographics [23].

### Distress versus depression

Modern fathers have increased expectations and responsibilities during the postpartum period than ever before. Fathers now share involvement with childcare, housework and are still income providers, changing their previous social role. The studies indicate that EPDS may actually be measuring a condition similar to general distress, including depressive and anxious symptoms, such as a state of worry or unhappiness [15, 17, 22]. The findings from Swedish fathers indicate that distress is more common than depression during the 3 to 4 month postpartum period. Fathers have reported difficulty meeting their day-to-day life needs, challenged with balancing family life and work demands. This leads to feelings of helplessness, anxiety, or irritability that more closely resemble distress disorder rather than depression.

Similar findings were also found in Chinese fathers. Lai et al. found that regardless of the depression diagnosis in the fathers, the participants most often reported being overwhelmed and blaming themselves when making mistakes [17]. Wong et al. determined that the help-seeking rate for depression and other emotional distress issues was lower in Chinese men than women due to societal norms of masculinity [25]. The Chinese society does not socially recognize men as husbands or fathers. Paternity leave or shorter working hours directed at assisting the family dynamic is not granted to new fathers. Many new fathers experience exhaustion and sleep deprivation leading to potential psychological breakdowns [17, 19]. It is apparent that the structure of assessing postpartum men and women is different, as it should be. Men reported more symptoms similar to general distress such as irritability, feelings of being overwhelmed, and/or aggressiveness that may be under detected on depression screenings, such as with the EPDS [15]. Swedish studies found that participants with possible depression were detected only by one of the two instruments used (EPDS or GMDS). Nishimura et al. studied Chinese fathers and reported the EPDS to be more accurate in detecting postnatal depression than the BDI, and the Patient Health Questionnaire (PHQ-9) [24]. The BDI is a self-report rating inventory that measures characteristic attitudes and symptoms of depression, while the PHQ-9 is a questionnaire that screens for the presence and severity of depression [24].

This suggests that the EPDS and GMDS probably identify different components of depression. Indicating that neither scale alone is optimal for adequate detection of paternal depression, as they measure different symptoms. The GMDS instrument may be capable of detecting distress of a longer duration than the EPDS due to its items asking about aggressiveness, outward reactivity, difficulties keeping self-control, and irritability. It may be reasonable to utilize a lower score with the EPDS to detect minor depression, but the adaptation of additional questions should be included to assess for general distress during the extended postpartum period.

### Limitations

For the clinician seeking to operationalize the findings of this work, there are limitations in interpreting the existing body of literature surrounding the screening of fathers for perinatal depression. An analysis of the available articles reveals significant variation in cutoff points among different cultures. For the authors, who practice in the United States, there is no available literature using American fathers as a study population, so an optimal cutoff point for use is unknown. An understanding of which items are most culturally appropriate is imperative. Localzo et al. noted that among Italian fathers, the items pertaining to crying and self-harm were very rarely endorsed [26]. Not all studies reported an item analysis with this level of granularity, limiting the ability to use these studies to inform future work.

Though many studies have examined the EPDS, alternative scales used were inconsistent across studies. The most common scales used in the United States, the PHQ-9 and Generalized Anxiety Disorder Scale have been minimally studied in regards to postpartum depression in men. An additional challenge is the paucity of analysis related to demographic characteristics of participating fathers and inconsistency in the time of screening administration. Only the articles by Carlberg et al. [15], Lai et al. [17], and Nishimura et al. [24] offered a substantial analysis of demographic factors that may impact instrument sensitivity. Others offered limited demographic reporting. Given the significant differences noted in positivity on various instruments by Carlberg et al. [15], additional work examining demographic factors associated with risk would be helpful. This information would be imperative for clinicians who may be wanting to identify at-risk groups and for appropriate instrument selection.

Participant recruitment was also a noted limitation in a number of studies. In the works by Edmondson et al and Nishimura et al. [20, 24], fathers were recruited through maternal screening. If mothers did not screen positively or did not elect to participate in the research, fathers would not have been presented with an opportunity to be a part of the research process. This may have an impact on prevalence data.

While universal screening of fathers is the consensus recommendation of the reviewed studies, there is no specific guidance regarding the timing of screening related to the sensitivity of the instruments studied. The article by Edmundson et al. also acknowledged timing as a potential limitation, noting that in their study, there were roughly 5 weeks that elapsed between survey screening with the EPDS and structured clinical interviews [20]. With each of the instruments examined, clear reporting of the timing of administration is of great importance. Clinician consumers of this research who are working with new fathers need to have a clear understanding of not only which tool to choose for screening their populations, but also when to administer it.

The authors acknowledge limitations within this review as well. As this review included only articles published in English, there may be pertinent literature that exists that has not been represented in this synthesis.

## Implications

### Future work

The study of this clinical problem is well suited for nursing research, particularly for the Doctor of Nursing Practice, as this practice gap is of direct relevance to the care of the childrearing family. Future research should include evaluating the prevalence of mood disorders in new American fathers, specifically generalized distress, anxiety, and depression. Future research should also examine the consistency between positive screening findings across common psychological clinical screening measures. Identification of the most sensitive screening measures for the detection of mood disorders in American fathers is needed. Development of a new tool may be needed to effectively assess and detect mood disorders in new fathers, if a new tool is indicated, validation of this tool will be necessary. After identification of the most sensitive screening measure has been established, identifying the appropriate screening interval for new American fathers will be warranted. After future data and consensus have been established, further research should be directed towards evaluating underrepresented and/or at-risk fathers such as LGBTQIA+, African American, and those who have adopted or fostered. Finally, the most appropriate health care provider to implement and utilize the screening measure in clinical practice will need to be identified to ensure proper and continued assessment is occurring for new American fathers.

### Suggested clinical practice

Currently, the NPA encourages screening fathers for depression at least twice during the first postpartum year [2]. This recommendation does not explicitly state which tool should be used for screening and when the screening should occur within the first year. Based on the most up to date research provided in this integrative review, it could be suggested to use the EPDS with a cut off score of 10 to screen new fathers for a mood disorder [15, 17, 20, 22, 26]. If the aim of screening new fathers is to prevent depression, the low cut off score of 10 could decrease the risk of missing cases of minor depression, which could lead to major depression in the new fathers [15]. However, the American Family Physician guidelines accept the PHQ-2 as an initial screening tool in all age groups. If depression is identified, the PHQ-9 or a clinical interview should be completed [27].

## Conclusion

To promote optimal outcomes for neonates, health care providers must assess the mental health and adaptation of all new parents, regardless of gender, and make appropriate referrals as needed. Additional research is needed to facilitate optimal care of the new family in regards to paternal mental health. The findings of these studies suggest further attention and research should be focused on developing screening of new fathers with a questionnaire based on combined scales in order to increase the detection of mood disorders, including generalized distress, anxiety, and depression. Research has found that cultural pressure may cause men to feel as though they are weak or less manly if they show signs of despair or self-doubt. As a result of these cultural standards, symptoms of depression may be disguised as anger or irritability. Future research should be directed towards validating a tool to assess general mood disorders, such as distress, depression, and anxiety, in American fathers while analyzing appropriate screening intervals during the postpartum period.
